# Haff Disease in Salvador, Brazil, 2016-2021: Attack rate and detection of toxin in fish samples collected during outbreaks and disease surveillance

**DOI:** 10.1016/j.lana.2021.100092

**Published:** 2021-11-01

**Authors:** Cristiane Wanderley Cardoso, Monaise Madalena Oliveira e Silva, Antônio Carlos Bandeira, Renan Bispo Silva, Ana Paula Pitanga Barbuda Prates, Ênio Silva Soares, José Jorge Moreno Silva, Lázaro José Rodrigues de Souza, Mirela Maisa da Silva Souza, Marcela Almeida Muhana, Rosildete Silva Santos Pires, José Fernando Araujo Neto, Manuela Sampaio Souza Santos, Luiz Laureno Mafra Junior, Thiago Pereira Alves, Mathias Alberto Schramm, Guilherme Sousa Ribeiro

**Affiliations:** a Secretaria Municipal de Saúde de Salvador, Salvador, Brazil; b Instituto Gonçalo Moniz, Fundação Oswaldo Cruz, Salvador, Brazil; c Secretaria de Saúde do Estado da Bahia, Salvador, Brazil; d Centro de Estudos do Mar, Universidade Federal do Paraná, Pontal do Paraná, Brazil; e Instituto Federal de Santa Catarina, Campus Itajaí, Itajaí, Brazil; f Faculdade de Medicina, Universidade Federal da Bahia, Salvador, Brazil

**Keywords:** Rhabdomyolysis, Haff disease, foodborne disease, palytoxin, public health, global health, outbreak, surveillance, epidemiology

## Abstract

**Background:**

From late 2016 to early 2021, cases of Haff disease, a rare cause of rhabdomyolysis, possibly due to poisoning by palytoxin-like compounds in seafood, were detected in Salvador, Brazil. Surveillance was established to detect additional cases aiming at describing the clinical characteristics of the cases, identifying associated factors, estimating disease attack rate, and investigating the presence of biotoxins and trace metals in selected fish specimens obtained from cases.

**Method:**

Between December/2016-January/2021, surveillance investigated Haff disease suspected cases, and obtained clinical and fish samples to test.

**Findings:**

Of 65 cases investigated during the 2016-2017 outbreak, 43 (66%) had high creatine phosphokinase (CPK) levels. Among those with laboratory-confirmed rhabdomyolysis, 38 (88%) were hospitalized, 11 (26%) required intensive care, and three (7%) dialysis. Ingestion of marine fish 24h before disease onset was reported by 74% of the cases with elevated CPK and by 41% of those without CPK measurement (P=0·02). Attack rate for individuals who ate fish related to the outbreak was 55%. Following this outbreak, surveillance identified 12 suspected cases between 2017-2019, and a second outbreak in 2020-2021, with 16 laboratory-confirmed rhabdomyolysis patients (five required intensive care; one died). No traces of ciguatoxins and metals were detected in fish specimens obtained in 2016, found to be *Seriola rivoliana*. Some fish samples from 2020 were screened for palytoxin (PlTX)-like compounds and contained detectable levels of molecule fragments characteristics of isobaric PlTX, ovatoxin-a (OVTX-a), OVTX-b and OVTX-d.

**Interpretation:**

These findings support the hypothesis that compounds related to PlTX accumulated in marine fish may be the toxic agent causing the disease. Haff disease is a life-threatening condition, requiring clinical suspicion for patients with sudden-onset myalgia following fish ingestion. Suspected cases should be reported to health authorities for investigation.

**Research in context**

**Evidence before this study**

Haff disease is a rare and potentially life-threatening cause of rhabdomyolysis that typically develops within 24 hours after ingestion of certain types of fish or crustacean. It has been postulated that the disease is caused by heat-stable toxins produced by microalgae and other marine invertebrates found in coral reefs and accumulated in the bodies of fishes and crustaceans by trophic transfer through the food chain. Haff disease was first described in 1924, near the Haff shores, East Prussia, and since then over 1,000 cases of the disease have been reported in Europe. Cases have also been detected in other regions of the world, including the United States of America and China. In South America, cases have recently been reported in Brazil (in the Amazon region and in the northeastern states). We used the term “Haff disease” to search MEDLINE and found only 92 articles for the period between 1948 and June, 2021; 36 of them published in the last ten years. Most publications are case series, outbreak reports, or review articles. Prospective surveillance investigations, designed to detect Haff disease cases and outbreaks over time, are scarce. Fewer than five studies have estimated the attack rate associated with sharing seafood meals implicated in the occurrence of Haff disease, all conducted in China. Furthermore, few studies have evaluated the presence of biotoxins in specimens of fish or crustaceans and seafood meal remains obtained from cases.

**Added value of this study**

In response to the first outbreak of Haff Disease in Salvador, northeastern Brazil, prospective surveillance was established to detect additional outbreaks or sporadic cases in the city. During December 2016 and January 2021, surveillance investigated patients who manifested sudden-onset muscle pain of unknown cause in association with elevated levels of creatinine phosphokinase (CPK) or with compatible symptoms, but for whom CPK measurement was not possible. The results provided a comprehensive description of the natural history of the disease, including an estimate of the attack rate associated with sharing a fish meal involved in the occurrence of Haff disease cases; characterization of clinical manifestations and outcomes of the disease, such as hospitalization rates, need for intensive care, and dialysis requirement; and identification of fish intake within 24 hours of the onset of symptoms as a factor associated with the diagnosis of the disease. In addition, laboratory assessment of biotoxins and trace metals in selected fish specimens and meal remains obtained from cases revealed the presence of palytoxin (PlTX)-like compounds, such as isobaric PlTX, ovatoxin-a (OVTX-a), OVTX-b and OVTX-d, but not of ciguatoxins and metals.

**Implication of all the available evidence**

Our study further supports the hypothesis that palytoxin-like compounds accumulated in the body of fish and crustaceans are the most likely toxic agent associated with Haff disease. Given the increase in the number of case reports of Haff disease in the last decade and the growing evidence that outbreaks can happen worldwide, including the American continent, our results reinforce that Haff disease should be considered as a potential cause of rhabdomyolysis for all patients with symptoms onset within 24 hours of ingestion of seafood or freshwater fish. In addition, our findings highlight the importance of reporting suspected cases to health authorities and maintaining local level surveillance for prompt investigation of reported cases.

Alt-text: Unlabelled box

## Introduction

Rhabdomyolysis is a syndrome caused by direct or indirect muscle injury, resulting in elevation of serum creatine phosphokinase (CPK) levels. As the kidneys eliminate muscle metabolites, urine may become darker presenting reddish to brown coloration.^1^ Haff disease is a rare cause of rhabdomyolysis that typically develops 24 hours after ingesting certain fish and crustacean. The rapid onset of disease after consumption of these cooked foods suggests that heat-stable toxins are the cause of the disease.^2^^,^^3^ Although the origin and type of toxins involved remain unclear, it is assumed that fish and crustaceans do not produce the toxins themselves, but rather accumulate compounds produced by other organisms – likely algae – via trophic transfer over the food web.

The disease was first described in 1924, near the Haff shores along the Baltic Coast in East Prussia. Since then, over 1,000 cases of Haff disease were reported in Europe.^4^ In 1984, cases of rhabdomyolysis were also detected in the United States of America, following consumption of different species of fish, mainly Buffalo fish (*Ictiobus cyprinellus*).^2^^,^^5^ Outbreaks have also been identified in China in 2000, with additional cases reported between 2009-2016.^6^ In Brazil, an outbreak of Haff disease involving 27 cases occurred in the Amazon region between June and September 2008.^7^ Most cases were identified in the city of Manaus and all patients were hospitalized. Three local freshwater fishes (“pacu”, *Mylossoma* spp.; “tambaqui”, *Colossoma macropomum*; and “pirapitinga”, *Piaractus brachypomus*) were implicated as the source of the outbreak at that opportunity.

In December 2016, the Centre for Strategic Information and Health Surveillance (CIEVS) of Salvador, Brazil was informed about nine patients who had attended an emergency health unit with clinical manifestations consistent with Haff disease. Cases continued to be reported in the city during the following weeks, totalizing 65 suspected cases until April 2017. We had previously described 15 of these cases.^8^ Salvador, the capital of the state of Bahia, is the fourth largest city in Brazil, located along the coast of the Northeast region of the country. The city has 80 Km of beaches bordering the Atlantic Ocean and the largest bay in the country, the Baía de Todos os Santos [All Saints Bay]. Moreover, the state of Bahia has the longest coastline in Brazil. However, finfish aquaculture production in Bahia is small, representing less than 3% of the national production in 2019^9^, and part of the fish consumed in the region comes from other states.

Here, we considerably expand the findings for the 2016-2017 outbreak, investigating cases’ clinical characteristics and associated factors, and estimating disease attack rate related to sharing fish meals with cases during the outbreak. Since then, CIEVS maintained surveillance and continued to detect sporadic cases, as well as a new outbreak in 2020-2021, which are also described herein. Furthermore, we also investigate the presence of biotoxins and trace metals in selected fish specimens and meal remains obtained from cases. Our findings should contribute to the limited literature on Haff disease in the Americas and help describe both the epidemiology and the possible role of toxins in the pathogenesis of the disease.

## Methods

### Case detection during the 2016-2017 outbreak

Based on the first reports of suspected cases of rhabdomyolysis, in December 2016, CIEVS issued an epidemiological alert (http://www.cievs.saude.salvador.ba.gov.br/surto-de-mialgia/) to all health units of Salvador, informing about a possible outbreak, asking clinicians to report cases of sudden-onset myalgia of unknown cause, and requesting a retrospective evaluation of medical records aiming to search for cases exhibiting compatible symptoms in the preceding month, which could have gone unrecognized.

The following case definition was used for case detection and reporting: “patients exhibiting (i) sudden-onset muscle pain of unknown cause, especially in the neck/trapezium region, associated to pain in other body regions (e.g., upper limbs, lower limbs, back region, thorax, and abdominal region), and (ii) elevated levels of creatinine phosphokinase (CPK), or compatible symptoms but from whom CPK measurement was not possible”. Reported cases with residence outside of Salvador were informed to the epidemiological office of their respective municipalities.

All patients identified as suspected cases were interviewed to collect data on demographics (age, sex, skin colour), clinical manifestations (date of onset of symptoms, signs and symptoms, hospitalization), and epidemiological exposures (history of fish ingestion and time to onset of symptoms, contact with animals and rainwater, meal in restaurants, use of illicit drugs within 24 hours prior to the onset of symptoms, use of medication within 48 hours prior to the onset of symptoms, physical exercise within 72 hours prior to the onset of symptoms, travel in the week before onset of symptoms, and vaccination within 15 days before onset of symptoms). We also investigated unreported individuals who fulfilled the case definition and consumed the same fish meal as a reported case. Data on clinical outcomes (dialysis, intensive care support, and death) and laboratory results were obtained from medical records.

### Epidemiological surveillance following the 2016-2017 outbreak

Following the 2016-2017 outbreak, Salvador health units continued to report suspected cases to CIEVS, using the aforementioned case definition. CIEVS investigated the reported cases, obtaining data on demographics, clinical manifestations and outcomes, and history of fish ingestion and time to onset of symptoms.

### Laboratory assessments

Patients’ samples of blood, faeces, and urine were collected for laboratory exams (bacterial culture and enterovirus testing). Whenever available, samples of the fish consumed by the cases were also collected for testing by the state public health laboratory and associated laboratories. From the samples obtained during the 2016-2017 outbreak, fish testing followed the American Public Health Association (APHA) standard methods^10^ for measurement of the total count of coliforms, and for detection of *Salmonella* sp., coagulase positive *Staphylococci* sp., *Bacillus cereus*, sporulated sulphite-reducing anaerobes, *Vibrio parahaemolyticus*, as well as to determine fish colour, odour, and aspect.

A sample of uncooked fish (“olho de boi”, *Seriola* sp.) from 2016 was obtained from a cluster of two cases that reported having acquired it in the main fish market of the city (the first had laboratory evidence of rhabdomyolysis and the other one did not have CPK levels measured). The sample was assessed by inductively coupled plasma mass spectrometry (ICP-MS) for quantification of metals (total arsenic, cadmium and lead), and by high performance liquid chromatography (HPLC) coupled to ICP-MS for arsenic speciation (certified reference material: Fish Muscle ERM BB422) at Adolf Lutz Institute (São Paulo, Brazil) and Federal University of ABC (Santo André, Brazil), respectively.^11^^,^^12^

Identification of fish species was performed according to the Centre for Food Safety and Applied Nutrition Standard Operating Procedure by the Food and Drugs Administration (FDA), in Alabama, USA, in the same sample evaluated for the presence of metals and in two additional uncooked samples from 2016, obtained from a cluster of three cases (one with elevated CPK levels and the others without CPK measurement) and from an isolated case (with high CPK levels).^13^ FDA also investigated these fish samples for the presence of the algal toxin ciguatoxin using both liquid chromatograph coupled with triple quadrupole mass spectrometry (LC-MS/MS) and in vitro neuroblastoma (N2a cells) cytotoxicity assay (CBA-N2a).

Another six extra samples of fish obtained in 2020 were examined by LC coupled to tandem MS (LC-MS/MS) at the Laboratory of Harmful Algae and Phycotoxins, Federal Institute of Santa Catarina (Itajaí, Brazil) for the presence of palytoxin-like compounds. Of these six samples, two (one fresh and one cooked, leftover from a meal) were obtained from a cluster of two cases, both with laboratory evidence of rhabdomyolysis; two (one fresh and one cooked) were obtained from an isolate case with high CPK levels; and the last two were fresh samples obtained from a local fish shop where some patients had purchased fish. Using multiple reaction monitoring (MRM), samples were screened for the presence of MS/MS transition ions characteristics of isobaric palytoxin (p-PlTX) and its analogues, the ovatoxins (OVTX), following a method adapted from Brissard et al. (2015).^14^ In addition, sample toxicity was evaluated using a 48-hour Swiss-mouse bioassay protocol for lipophilic biotoxins.^15^

Finally, potable water samples were collected at the four water treatment stations that supply Salvador in December 2016, and the samples were sent to the State Central Laboratory of Pernambuco to test for cyanobacterial and cyanotoxins levels.^16^

### Statistical analysis

Descriptive analysis included frequencies, medians, and ranges or interquartile ranges. Fisher's exact test was used to compare the frequencies of potential risk exposures between suspected cases with laboratory-confirmed rhabdomyolysis and suspected cases that did not measure CPK levels; a significant difference was defined by a two-tailed P-value <0.05. Attack rate for individuals who ate fish related to the 2016-2017 Haff disease outbreak was calculated by dividing the number of cases simultaneously reporting fish ingestion and fulfilling the case definition of suspected Haff disease by the total number of subjects who ate fish related to occurrence of one or more cases, multiplied by 100. For this analysis, we considered as suspected Haff disease cases those patients with increased CPK levels, or with unmeasured CPK but with a fish meal shared with a case exhibiting increased CPK levels.

### Ethics statement

This investigation was performed as part of the routine surveillance activities of Salvador CIEVS. All study participants provided verbal consent before being interviewed. Data analysis was performed using de-identified data. The Swiss-mouse bioassay followed national standards for the care and use of animals in research, in accordance with the protocol approved by the Ethical Committee for Animal Use of Universidade do Vale do Itajaí (CEUA – UNIVALI 047/11).

### Role of the funding source

No specific funding source was required for completion of this work, which was conducted with the support of public institutions to which the authors are affiliated. These institutions had no role in study design, data collection, data analysis, data interpretation, or manuscript preparation. All authors approved the manuscript before submission.

## Results

### Outbreak investigation, 2016-2017

#### Clinical characteristics

**Figure 1:**
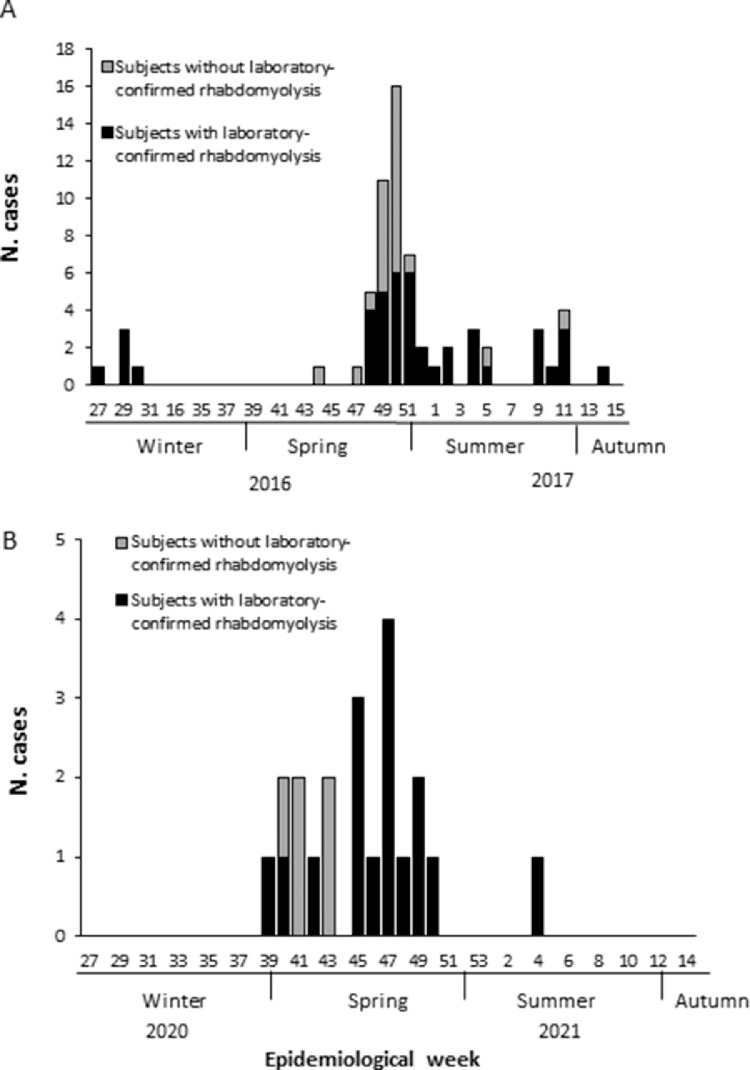
**Number of Haff disease cases detected during outbreaks in (A) 2016-2017 and (B) 2020-2021, according to epidemiological week of symptom onset and laboratory status, Salvador, Brazil.** Cases with and without laboratory-confirmed rhabdomyolysis are those with raised CPK levels and those for which CPK levels were not measured, respectively.

Of 78 reported cases during the 2016-2017 outbreak, 13 were excluded from the investigation because of absence of myalgia (4 subjects), normal CPK levels (6), medical assistance in another city (2), or onset of symptoms in another city (1). Of the 65 cases investigated, 43 (66%) had laboratory-confirmed rhabdomyolysis, temporally distributed between July 2016 and April 2017 (peak in December 2016) (Figure 1A). Patients’ demographics and clinical characteristics are described in Table 1. Despite the short period of illness, disease severity was high, leading 88% of confirmed cases to hospitalization, 26% to admission to intensive care unit (ICU), and 7% to dialysis (Table 1). Laboratory findings commonly observed among cases with confirmed rhabdomyolysis are described in Table 2. The highest CPK levels observed during the disease course ranged from 291 to 113,639 U L^−1^ (median: 12,433 U L^−1^).

**Table 1:** Demographic and clinical characteristics of suspected Haff disease cases during an outbreak in Salvador, Brazil, according to laboratory confirmation of rhabdomyolysis, 2016-2017 (N=65)

**Characteristics**	**Subjects with laboratory-confirmed rhabdomyolysis(N = 43)**	**Subjects without laboratory-confirmed rhabdomyolysis(N = 22)**
	**Number/response (%) or median [IQR]**	
**Demographics**		
Age, years	42 [30-62]	42 [32-51]
Female	22/43 (51)	16/22 (73)
**Skin color**		
White	16/41 (39)	9/20 (45)
Mixed	14/41 (34)	6/19 (32)
Black	11/41 (27)	6/19 (32)
**Clinical** **manifestations**		
Muscle pain	43/43 (100)	22/22 (100)
**First affected region/muscle**		
Trapezius	22/41 (54)	14/22 (64)
Lower limbs	6/41 (15)	2/22 (9)
Latissimus dorsal	2/41 (5)	0/20 (0)
Upper limbs	2/41 (5)	3/22 (14)
Abdomen	1/41(2)	1/22 (5)
Others	8/38 (21)	2/22 (9)
Dark urine	20/41 (49)	7/22 (32)
Muscle pain at touch	19/42 (45)	10/22 (46)
Dry mouth	13/42 (31)	8/22 (36)
Arthralgia	12/42 (29)	12/22 (55)
Headache	12/43 (28)	13/22 (59)
Dyspnea	11/42 (26)	7/22 (32)
Fever	5/42(12)	2/22 (9)
Vomit	5/42 (12)	3/22 (14)
Cough	5/42 (12)	4/22 (18)
Pruritus	4/42 (10)	8/22(36)
Retro-orbital pain	4/42 (10)	4/22 (18)
Diarrhea	4/42 (10)	7/20 (35)
Conjunctivitis	3/42 (7)	3/22 (14)
Adenopathy	3/42 (7)	2/22 (9)
Exanthema	2/42 (5)	7/22 (32)
**Disease outcomes**		
Number of days of illness	3 [2-5]	3 [2-7]
Search for medical care a	42/43 (98)	15/22 (68)
Hospitalization	38/43 (88)	3/20 (15)
Intensive care unit admission	11/43 (26)	0/21 (0)
Dialysis	3/42 (7)	0/22 (0)

a One laboratory-confirmed rhabdomyolysis case did not seek for medical care but performed CPK testing.

**Table 2:** **Initial and maximal values for laboratory tests among** suspected Haff disease **cases** with laboratory-confirmed rhabdomyolysis**,** during an outbreak in Salvador, Brazil, **2016-2017 (N=43)**.

**Laboratory exam**	**N**	**Initial value**	**Maximum value**	**Normal range**^a^
		**Median (min-max)**		
CPK (U L^−1^)	43	1,369 (78·0-62,654)	12,433 (291·0-113,639)	30-170
AST (U L^−1^)	32	168·5 (26·0-1,325))	316·0 (40-2,403)	0-35
ALT (U L^−1^)	30	88·5 (20·0-683·0)	156·0 (20·0-862·0)	0-35
Creatinine (mg dL^−1^)	29	0·7 (0·3-8·1)	0·9 (0·3-9·7)	0·7-1·3
Urea (mg dL^−1^)	30	22·5 (11-196)	30·0 (11-227)	17-43
Total bilirubin (mg dL^−1^)	18	0·7 (0·3-1·3)	0·7 (0.5-1·5)	0·3-1·2
Direct bilirubin (mg dL^−1^)	16	0·3 (0·0-0·5)	0·4 (0·0-0·7)	0-0·3
Indirect bilirubin (mg dL^−1^)	16	0·4 (0·0-0·8)	0·5 (0·0-1·3)	0·3-0·9
Hemoglobin (g dL^−1^)	31	13·3 (8·8-17·0)	14·3 (31·3-48·6)	12-17^b^
Hematocrit (%)	29	39·4 (26·8-48·6)	42·0 (31·3-48·6)	36-51^c^
Leucocyte count (cells mm^−3^)	31	7,400 (1,777-24,400)	8,660 (1,770-24,400)	4,500-10,000
Platelets (x10^3^ µL^−1^)	27	210 (130-278)	220 (130-398)	150-350

a Source: Normal laboratory values in healthy adults (https://www.msdmanuals.com/professional/resources/normal-laboratory-values/normal-laboratory-values).

b Range for female: 12-16 g dL^−1^; Range for male: 14-17 g dL^−1^.

c Range for female: 36-47%; Range for male: 41-51%.

Reported patients who did not have CPK levels measured less frequently reported dark urine compared to those with elevated CPK levels, and more frequently reported arthralgia, headache, exanthema, pruritus, and diarrhea (Table 1). In addition, their clinical outcomes were much less severe: only 15% were hospitalized and none required ICU admission or dialysis (Table 1), suggesting that they had a milder form of Haff disease or, most likely, their illness had another etiology.

#### Epidemiological risk exposure

The most frequent epidemiological exposure among the rhabdomyolysis-confirmed cases was fish ingestion prior to symptoms onset; 36 (84%) reported fish ingestion in the previous 72 hours (Table 3). Of these 36 cases, 14 (40%) and 11 (31%) reported eating the saltwater fishes “olho de boi” (*Seriola* sp.) and “badejo” (*Mycteroperca* sp.), respectively, and four (11%) reported eating both. Another five cases did not know the type of fish eaten, one reported eating the freshwater fish “tilápia” (*Tilapia* sp.), and one reported eating – 24 hours before symptom onset – a local Afro-Brazilian food that might contain fish by-products in its preparation.

**Table 3:** Frequency of epidemiological exposures of suspected Haff disease cases during an outbreak in Salvador, Brazil, according to the presence of laboratory-documentation of rhabdomyolysis, 2016-2017 (N=65)

**Exposures**	**Subjects with laboratory-confirmed rhabdomyolysis(N = 43)**		**Subjects without laboratory-confirmed rhabdomyolysis(N = 22)**		***P* value**
	**Number/response (%)**				
Ingestion of fish (≤72 hours)		36/43 (84)	13^b^ /22 (59)		0·06
Ingestion of fish (≤24 hours)		32/43 (74) a	9/22 (41)		0·02
Contact with animals (past 24 hours)		19/42 (45)	10/22 (46)		1·00
Travel (past 7 days)		13/42 (31)	8/21 (38)		0·77
Physical exercise (past 72 hours)		10/42 (24)	5/22 (23)		1·00
Use of medications (past 48 hours) c		10/43 (23)	6/22 (27)		0·95
Meal at restaurant (past 24 hours)		9/40 (23)	6/22 (27)		0·90
Use of illicit drugs (past 24 hours)		3/42 (7)	1/21 (5)		1·00
Contact with rainwater (past 24 hours)		1/42 (2)	1/22 (5)		1·00
Vaccination (past 15 days)		0/42 (0)	0/21 (0)		NA

NA: Not available.

a The group laboratory-confirmed rhabdomyolysis cases included one patient who reported, within 24 hours before the symptoms onset, ingestion of Afro-Brazilian food, which might have fish by-products in its preparation.

b The group without laboratory-confirmed rhabdomyolysis cases included one subject who reported consuming fish, but was unable to determine the timing before symptoms onset. We considered this case as having eaten fish in the past 72 h before symptoms onset, but not in the past 24 hours.

c Medications: ketoprofen, losartan, levothyroxine, olmesartan, pantoprazole, amlodipine besylate, aululpride, D-vitamin, statin, sulfamethoxazole-trimethoprim, metoprolol succinate, orphenadrine, and acetylsalicylic acid.

Frequencies of potential risk exposure were similar between reported cases with and without laboratory-confirmed rhabdomyolysis, except by the ingestion of fish in the previous 24 hours, reported by 74% of the rhabdomyolysis-confirmed cases and 41% of the non-confirmed cases (P = 0·02) (Table 3). Of the 36 rhabdomyolysis-confirmed cases who reported eating fish within 72 hours prior to the onset of symptoms, 34 remembered the time of fish consumption and the time of symptoms onset, allowing estimation of a median incubation period of 10 hours (IQR: 6-24 h). Of note, of these 36 cases, 31 (86·1%) prepared the fish at home, two (5·6%) consumed the fish in the home of relatives, two (5·6%) in restaurants, and one (2·8%) on the beach.

#### Attack rate associated with fish consumption

By interviewing the rhabdomyolysis-confirmed cases, we discovered whether other individuals had eaten the same suspected fish or fish-based meal, and we contacted these individuals to verify if they had developed muscle pain. This allowed us to estimate the attack rate associated with sharing a fish meal related to the outbreak at 55% (table 4). Noteworthy, 25 (69%) of the 36 confirmed cases with a history of prior fish ingestion clustered into 12 groups, consisting of two to four subjects who reported sharing the same fish meal (Table 4).

**Table 4:** Attack rate of Haff disease among clusters of individuals who reported ingestion of a same fish as a case during the 2016-2017 outbreak in Salvador, Brazil.

**Cluster**	**Number of Haff disease suspected cases among clusters of people sharing a fish meal**^a^	**Number of Haff disease suspected cases and their contacts sharing a fish meal**^b^	**Attack rate (%)**
A	4	4	100
B	2	4	50
C	3	7	43
D	2	2	100
E	3	6	50
F	3	8	38
G	4	4	100
H	2	4	50
I	2	2	100
J	2	11	18
K	2	5	40
L	2	2	100
M	1	4	25
N	1	2	50
O	1	2	50
P	1	5	20
Q	1	5	20
R	1	2	50
S	1	2	50
T	1	2	50
U	1	1	100
V	1	2	50
X c	1	1	100
**TOTAL**	42	87	55

a Of the 42 Haff disease suspected cases, 36 had laboratory-confirmed rhabdomyolysis and 6 did not have the CPK level measured, but belonged to a cluster that had at least one laboratory-confirmed cases.

b Number of Haff disease cases and case contacts sharing a fish meal in the past 72h that was related to Haff disease cases occurrence.

c This patient reported ingestion of an afro-brazilian food that might contain fish by-products within 24 h before symptoms onset. Because we cannot guarantee that he consumed fish, we performed a sensitive analysis, removing him from the attack rate calculation. The attack rate estimated in this sensitive analysis (48% (41/86)) did not differ substantially from the one average attack rate shown in the table.

#### Laboratory investigation of patients with Haff disease

As the laboratory assessment was performed as part of an outbreak investigation, tests were not systematically performed on all patients. Reverse transcription polymerase chain reaction (RT-PCR) for enteroviruses was performed in the serum of 13 cases, of which eight also had the test performed on stool samples. From those, 12 (92·3%) serum samples and four (50·0%) stool samples tested positive (11 and three of them with confirmed rhabdomyolysis, respectively). However, viral isolation (culture in HEP-2 and RD cells) was positive for enteroviruses in only one (11·0%) of eight stool samples and in none of the 13 serum samples. This single case with enterovirus-positive stool culture was a 10-year-old girl who had a maximum CPK level of 851·2 U L^−1^ but denied previous fish ingestion. Blood and stool cultures were performed in five and four of the 13 cases evaluated by RT-PCR for enteroviruses, respectively, and all samples tested negative for bacterial growth. Laboratory tests for cytomegalovirus, Epstein-Barr virus, eritrovirus, and echovirus were performed in samples from three patients with confirmed rhabdomyolysis and all of them were negative.

#### Analysis of fish and water samples

Three samples of fish obtained from different clusters of suspected cases were analysed to identify the fish species. Two were identified as *Seriola rivoliana* (common names in Brazil: “olho de boi”, “olhete-bacamarte” and “arabaiana”). The third fish was identified as *Bartholomaei carangoides* (common names in Brazil: “garajuba” and “xaréu”).^17^ Although the reported case who consumed *B. carangoides* had a clinical presentation and epidemiological history compatible with Haff disease, her unique CPK measurement was within normal limits (56 U L^−1^), and she was ultimately excluded as a case in this outbreak.

These three fish samples underwent further microbiological analyses and were also tested for the presence of ciguatoxin, yielding negative results in all cases. In another fish sample, the concentrations of lead and cadmium were below the quantification limits (0·08 and 0·04 mg Kg^−1^) and the concentration of inorganic arsenic (48·1 µg Kg^−1^) was below the safety limits for human consumption.^18^ Finally, cyanobacterial and cyanotoxin levels, as determined in 31 potable water samples collected in the four water treatment stations supplying Salvador, were below regulatory levels according to the current legislation in Brazil.^16^

### Epidemiological surveillance, 2017-2019

#### Clinical characteristics

Following the 2016-2017 outbreak, Haff disease continued to be reported in Salvador. In September 2017, a cluster of two couples who developed myalgia after consumption of “olho de boi” were reported, but it was not possible to confirm whether they had increased CPK levels. One month later, two cases – a mother and her son – with the highest CPK levels of 40,000 and 24,894 U L^−1^, respectively, and a history of “olho de boi” fish consumption were reported. In December 2017, a male with a history of “badejo” fish consumption exhibited high CPK levels (maximum of 18,717 U L^−1^). In September 2018, other three cases were reported: a couple with the highest CPK levels of 4,404 and 9,908 U L^−1^ and a history of “badejo” fish consumption; and a woman with history of consumption of an unknown fish (highest CPK level: 93,530 U L^−1^) who required intensive care support and dialysis. In September 2019, a couple who developed myalgia after consumption of "olho de boi” fish required hospitalization with high levels of CPK (29,998 and 26,743 U L-1).

### Outbreak investigation, 2020-2021

#### Clinical characteristics

In 2020, during the COVID-19 pandemic, there was a new increase in the number of reported patients suspected of Haff disease in Salvador: 21 patients (16 of them with laboratory-confirmed rhabdomyolysis) were detected between September 2020 and January 2021 (Figure 1B). The median age for the 16 cases with laboratory-confirmed rhabdomyolysis was 56·5 (min-max: 26-89) years. The median of the first and the maximum CPK measurements were 11,766 (min-max: 500 to >100,000) U L^−1^ and 26,246 (min-max: 500 to >100,000) U L^−1^, respectively. Darkness of urine was reported by nine (56%) of the 16 patients with increased CPK levels. All 16 cases received medical care, five (31%) required UCI admission, none had dialysis, and one (6%) died. Fifteen (94%) of them reported fish consumption before symptoms onset (14 developed symptoms within the first 24 hours after fish consumption; median time: 10 (min-max: 2-48) hours). “Olho de boi” was the most frequent ingested fish, reported by six (46%) of 13 cases who informed the type of consumed fish.

#### Investigation of palytoxin-like compounds in fish samples

Six samples of “olho de boi”, four of them consisting of *in natura* fish and two of cooked fish meals (fish stew), related to some of the Haff disease cases detected in 2020, were screened for the presence of palytoxin-like compounds. Traces of MS/MS transition ions characteristics of isobaric palytoxin (PlTX), OVTX-a, OVTX-b, and OVTX-d were detected in both samples of cooked fish. Aliquots from all six samples were tested in mouse bioassays, challenging groups of three to four Swiss mice. Only one sample of raw fish caused the death of one animal (from a group four challenged animals) within 12-24 hours following intraperitoneal injection. Even though, all other tested animals were fully recovered after 48 hours and no detectable levels of isobaric PlTX or OVTX were found by LC-MS/MS in the sample associated with the single mouse death.

## Discussion

Our study provided detailed clinical characterization of Haff disease cases, identified fish consumption within 24 hours of symptoms onset as an associated risk exposure, estimated disease attack rate after ingestion of a fish related to the occurrence of cases in 55%, implicated the saltwater fish species *S. rivoliana* as the likely source of contamination, and detected palytoxin-like compounds in fish specimens consumed by some of the cases, suggesting that they may play a role in disease pathogenesis.

Because Haff disease clinical presentation is not specific and some patients evolve with milder symptoms, we may have lost detection of additional cases who did not seek medical care or who were misdiagnosed for other diseases. Conversely, we cannot rule out that some of the suspected cases, especially those without CPK measurement, had another cause of myalgia, such as infection by dengue, chikungunya, or Zika viruses, which have been concomitantly transmitted in Salvador since 2015,^19^, ^20^, ^21^ or epidemic myalgia^22^ caused by enteroviruses, such as echovirus 22 and 23, or by human parechoviruses (HPeV).^23^ In addition, we cannot discard that some cases with elevated levels of CPK, but denying fish ingestion, had another etiology for the rhabdomyolysis (e.g., leptospirosis).

In a previous case-series, comprising the first 15 cases detected during the 2016-2017 Haff disease outbreak in Salvador,^8^ RT-PCR analyses were run for chikungunya and Zika viruses, as well for enteroviruses and Parechovirus. All tested samples yielded negative results for most viruses, except enterovirus, for which non-specific products were amplified in four samples, but not confirmed after gene sequence analysis.^8^ During the current investigation, additional testing for enterovirus was performed. Although 16 of 21 samples of serum or faeces had been RT-PCR-positive for enterovirus, only one stool sample out of 21 faeces or serum sample undergoing viral isolation tested positive. Besides the discrepancy in the positivity for enterovirus between the RT-PCR and both the cell culture and gene sequencing,^8^ the clinical characteristics of our patients were not compatible with typical enterovirus infections. Moreover, the association with fish ingestion after cooking, which should kill viral pathogens, also suggests that an enterovirus was not the etiology for the cases described.

Ingestion of cooked seafood products 24 hours before symptoms onset has been identified as the culprit for Haff disease, with incubation periods generally ranging from 6 to 8 hours.^24^ During the 2016-2017 outbreak, 84% of the laboratory-confirmed cases reported fish ingestion 1 to 72 hours before initiation of symptoms (74% within 24 hours). During the 2020-2021 outbreak, 94% of the confirmed cases reported fish consumption 2 to 48 hours before symptoms onset (93% within 24 hours). The longer interval between fish ingestion and symptoms onset that we observed in some cases is likely due to erroneous recall and misinformation, because it was not always possible to interview the cases within a few days after disease onset. Noteworthy, by tracing unreported individuals who had shared a fish meal with a confirmed case, we could estimate the average risk of Haff disease development after sharing a fish meal related to Haff disease occurrence to be around 50%. Variations in the number of subjects involved in outbreaks are often reported in seafood-borne natural toxin poisoning^6^^,^^25^^,^^26^ and may be related to individual susceptibility or to inoculum dose. To date, only a few studies performed in China have attempted to estimate the attack rate of Haff disease among individuals who ate fish or crayfish meals implicated in the occurrence of cases.^6^^,^^27^^,^^28^ Thus, further outbreak investigations should also assess disease attack rate.

Most of the Haff disease cases detected reported eating either “olho de boi” (*Seriola* sp.) or “badejo” (*Mycteroperca* sp.). Genetic testing in fish samples obtained from two cases with laboratory evidence of rhabdomyolysis confirmed that “olho de boi” (*S. rivoliana*) was truly associated with the outbreak. “Olho de boi” had also been implicated as the origin of the disease by a couple who initiated myalgia within a few hours after consuming a fish bought during a travel to the northeast of Brazil.^29^ However, we cannot rule out the involvement of “badejo” (*Mycteroperca* spp.) in cases that occurred in Salvador, as only a small number of fish samples underwent genetic testing to define the species. “Olho de boi” and “badejo” are both carnivorous species and prey upon fish, crustaceans and mollusks living in offshore areas.

The sudden-onset of myalgia after consumption of cooked (or sometimes uncooked) fish or crayfish, and the resolution of the muscle pains within a few days following symptoms onset is highly suggestive of a disease pathogenesis involving ingestion of preformed, heat-stable toxins present in the fish.^2^^,^^3^^,^^30^ It has been postulated that Haff disease may be due to poisoning by palytoxin^24^ accumulated in seafood. Palytoxin and its analogues are extremely potent and complex non-protein toxins, produced by marine dinoflagellates of the genus *Ostreopsis*, by zoanthids (i.e., *Palythoa*), and possibly by cyanobacteria of the genus *Trichodesmium*,^31^ all vastly distributed in tropical and subtropical waters,^32^^,^^33^ including the coast of Brazil. Palytoxin-like compounds have been associated to human poisoning episodes, including fatal cases,^34^ even though their detection in seafood samples implicated in poisoning outbreaks is scarce so far. This may be explained by the difficulty in obtaining specimens to test, as well as by the lack of laboratory capabilities for proper investigation in most areas. Conversely, several previous studies evaluating samples of seafood consumed by Haff disease cases failed to detect different biotoxins, regardless of the test used.^2^^,^^23^^,^^35^^,^^36^

We analysed fish samples obtained from six cases reported in 2020. Detectable levels of isobaric palytoxin and ovatoxins were found in two cooked fish samples, but not in four *in natura* fish samples. In contrast, samples from three fishes collected during the 2016 outbreak were negative for the presence of ciguatoxins (produced by the dinoflagellate *Gambierdiscus* spp.), as well as pathogenic microorganisms and metals. Furthermore, cyanobacteria and cyanotoxins were not present at unsafe levels in potable water from main Salvador's suppliers. Altogether, these findings give support to the hypothesis that palytoxins are the most likely toxic agent linked to the development of Haff disease in Salvador.

Before the 1990s, most cases of Haff disease were described in Eastern Europe and Sweden.^3^ But since then, cases have been increasingly reported in other countries, especially China, the United States of America, and Brazil. Given that fish and seafood production has greatly increased in recent decades worldwide (from <40 million tons in 1961 to >150 million tons in 2013)^37^ and that per capita consumption of fish and seafood has followed the trend (increasing from 9 kg per capita per year in 1961 to 19 kg per capita per year in 2013),^38^ we can speculate that the number of Haff disease cases will continue to grow. However, social and cultural aspects have an important influence on regional consumption of fish and seafood. For example, in North, Central and South America, per capita consumption of fish and seafood was 21.6 kg, 9.1 kg, and 10.3 kg per year in 2013, respectively (in Brazil, it ranged between 9.0 kg and 10.9 kg per year between 2013 and 2017).^38^ Furthermore, the clinical manifestations of Haff disease are very nonspecific, requiring medical astuteness to suspect the diagnosis. Thus, it is likely that cases are reported more frequently in regions were consumption of fish and seafood is higher and where physicians are more familiar with the disease, such as in places where cases have already been detected. Because of the recent Haff disease outbreaks in the United States of America and Brazil, it is necessary to strength health surveillance and medical training for detection of Haff disease in American countries.

Despite the study limitations (i.e., underreporting or case misclassification; absence of a comprehensive diagnostic evaluation to discard other potential disease aetiologies for every case; only a small number of cases had remaining portions from the consumed fish for testing), our long-term surveillance and outbreak investigations provided novel insights in the understanding of Haff disease epidemiology and pathogenesis. Haff disease should be considered as a cause of rhabdomyolysis for all patients with symptoms onset within 24 hours of fish consumption. As there are no laboratory tests available for the diagnosis of Haff disease, a high level of medical suspicion is required, based on clinical, epidemiological and laboratory parameters. All suspected case must be reported to health authorities and local outbreak investigations must be carried out, as sporadic cases are relatively rare.

## Contributors

Conceptualization: CWC and GSR; investigation: CWC, ACB, APPBP, ESS, JJMS, LJRS, MMSS, MAM, RSSP, JFAN, MSSS, LLMJ, TPA, MAS; verification of the underlying data and data analysis: CWC, MMOS, RBS, and GSR; supervision: GSR; writing – original draft: CWC; writing – review & editing: all authors; approval of the final version: all authors.

**Haff Disease in Salvador, Brazil, 2016-2021: Attack rate and detection of toxin in fish samples collected during outbreaks and disease surveillance**

## Data sharing statement

Anonymous data will be made available for researchers who present a methodologically sound proposal upon reasonable request to the Centre for Strategic Information and Health Surveillance (CIEVS), Secretary of Health of Salvador, Brazil (http://www.cievs.saude.salvador.ba.gov.br/).

## Funding

There was no funding for this study.

## Declaration of interest

All authors have nothing to disclose.

## Appendix Supplementary materials

Image, application 1

## References

[1] Khan FY. (Oct 2009). Rhabdomyolysis: a review of the literature. Neth J Med.

[2] Buchholz U, Mouzin E, Dickey R, Moolenaar R, Sass N, Mascola L. Haff disease: from the Baltic Sea to the U.S. shore. Emerg Infect Dis. 2000 Mar-Apr;6(2):192-5. https://doi.org/10.3201/eid0602.000215. PMID: 10756156; PMCID: PMC2640861. 10.3201/eid0602.000215 PMC2640861 10756156

[3] Diaz JH. (2015 Mar 19). Global incidence of rhabdomyolysis after cooked seafood consumption (Haff disease). Clin Toxicol (Phila).

[4] Jeddeloh Zu, Haffkrankheit B. (1939). Erg Inn Med.

[5] Centers for Disease Control and Prevention (CDC). Haff disease associated with eating buffalo fish–United States, 1997. MMWR Morb Mortal Wkly Rep. 1998 Dec 25;47(50):1091-3. PMID: 9883771 9883771

[6] Chan TYK. (2016). The Emergence and Epidemiology of Haff Disease in China. Toxins.

[7] Dos Santos MC, Albuquerque BC, Pinto RC, Aguiar GP, Lescano AG, Santos JHA (2009). Outbreak of Haff disease in the Brazilian Amazon. Pan Am J Public Health.

[8] Bandeira AC, Campos GS, Ribeiro GS, Cardoso CW, Bastos CJ, Pessoa TL (December 2016). Clinical and laboratory evidence of Haff disease -case series from an outbreak in Salvador, Brazil. Euro Surveill.

[9] Ximenes LF. (Jan 2021). Fish Production in Brazil and Northeast Brazil [In Portuguese. Caderno Setorial ETENE.

[10] (2001).

[11] Batista BL, Nacano LR, De Souza SS, Jr Barbosa F (2012 Jan 18). Rapid sample preparation procedure for As speciation in food samples by LC-ICP-MS. Food Addit Contam Part A Chem Anal Control Expo Risk Assess.

[12] Batista BL, Souza JM, de Souza SS, Jr Barbosa F (2011 Jul 15). Speciation of arsenic in rice and estimation of daily intake of different arsenic species by Brazilians through rice consumption. J Hazard Mater.

[13] (2011). U. S. Food and Drug Administration. Single Laboratory Validated Method for DNA-Barcoding for the Species Identification of Fish.

[14] Brissard C, Hervé F, Sibat M, Séchet V, Hess P, Amzil Z (2015). Characterization of ovatoxin-h, a new ovatoxin analog, and evaluation of chromatographic columns for ovatoxin analysis and purification. J. Chromatogr. A..

[15] Riobó P, Paz B, Franco JM, Vázquez JA, Murado MA, Cacho E. (2008 Apr 25). Mouse bioassay for palytoxin. Specific symptoms and dose-response against dose-death time relationships. Food Chem Toxicol.

[16] Ministério da Saúde. (31 Mar 2021). Secretaria de Vigilância em Saúde. Portaria N°. 2.914, de 12 de dezembro de 2012.

[17] Froese R, FishBase Pauly D. (11 Feb 2021). World Wide Web electronic publication. 2020. www.fishbase.org, version (12/2020).

[18] (1995). Food and Agriculture Organization of the United Nations and World Health Organization. Codex Stam.

[19] Cardoso CW, Paploski IA, Kikuti M, Rodrigues MS, Silva MM, Campos GS (Dec 2015). Outbreak of Exanthematous Illness Associated with Zika, Chikungunya, and Dengue Viruses. Salvador, Brazil.

[20] Cardoso CW, Kikuti M, Prates APPB, Paploski IAD, Tauro LB, Silva MMO (2017). Unrecognized Emergence of Chikungunya Virus during a Zika Virus Outbreak in Salvador, Brazil. PLoS Negl Trop Dis.

[21] Silva MMO, Tauro LB, Kikuti M, Anjos RO, Santos VC, Gonçalvez TSF (2019). Concomitant Transmission of Dengue, Chikungunya, and Zika Viruses in Brazil: Clinical and Epidemiological Findings From Surveillance for Acute Febrile Illness. Clin Infect Dis.

[22] Mizuta K, Kuroda M, Kurimura M, Yahata Y, Sekizuka T, Aoki Y (2008). Epidemic Myalgia in Adults Associated with Human Parechovirus Type 3 Infection. Yamagata, Japan.

[23] de Crom SC, Rossen JW, van Furth AM, Obihara CC. (2016 May 7). Enterovirus and parechovirus infection in children: a brief overview. Eur J Pediatr.

[24] Langley RL, Bobbitt WH (Nov 2007). Haff disease after eating salmon. South Med J.

[25] Chan TY. (2015 Mar 2). Emergence and epidemiology of ciguatera in the coastal cities of southern China. Mar Drugs..

[26] Ma H, Wu J, Qin W, Lin C, Li D, Zha B (2016). Outbreak of Haff Disease along the Yangtze River. Anhui Province, China.

[27] Huang X, Li Y, Huang Q, Liang J, Liang C, Chen B (2013 May 6). A past Haff disease outbreak associated with eating freshwater pomfret in South China. BMC Public Health.

[28] Guo B, Xie G, Li X, Jiang Y, Jin D, Zhou Y (2018 Nov 17). Outbreak of Haff disease caused by consumption of crayfish (Procambarus clarkii) in nanjing, China. Clin Toxicol (Phila).

[29] Almeida LKR, Gushken F, Abregu-Diaz DR, Muniz R, LH Degani-Costa (2019 Jul 24). Rhabdomyolysis following fish consumption: a contained outbreak of Haff Disease in São Paulo. Braz J Infect Dis.

[30] Huang C, Peng L, Gong N, Xue C, Wang W, Jiang J (2019). A Retrospective Analysis of Crayfish-Related Rhabdomyolysis (Haff Disease). Emerg Med Int.

[31] Kerbrat AS, Amzil Z, Pawlowiez R, Golubic S, Sibat M, Darius HT (2011 Mar 31). First evidence of palytoxin and 42-hydroxy-palytoxin in the marine cyanobacterium Trichodesmium. Mar Drugs.

[32] Nascimento SM, França JV, Gonçalves JE, Ferreira CE. (2012 Apr 4). Ostreopsis cf. ovata (Dinophyta) bloom in an equatorial island of the Atlantic Ocean. Mar Pollut Bull.

[33] Tibiriçá CEJA, Leite IP, Batista TVV, Fernandes LF, Chomérat N, Herve F, Hess P, Mafra LL (2019 Jul 27). Ostreopsis cf. ovata Bloom in Currais, Brazil: Phylogeny, Toxin Profile and Contamination of Mussels and Marine Plastic Litter. Toxins (Basel).

[34] Alcala AC, Alcala LC, Garth JS, Yasumura D, Yasumoto T. (1988). Human fatality due to ingestion of the crab Demania reynaudii that contained a palytoxin-like toxin. Toxicon.

[35] Huang X, Li Y, Huang Q, Liang J, Liang C, Chen B (2013 May 6). A past Haff disease outbreak associated with eating freshwater pomfret in South China. BMC Public Health.

[36] Suzuki T, Watanabe R, Matsushima R, Ishihara K, Uchida H, Kikutsugi S (2013 May 28). LC-MS/MS analysis of palytoxin analogues in blue humphead parrotfish Scarus ovifrons causing human poisoning in Japan. Food Addit Contam Part A Chem Anal Control Expo Risk Assess.

[37] Ritchie H, Roser M (2019). Published online at OurWorldInData.org. Seafood and fish production.

[38] Ritchie H (Jul 2021). Roser M. Seafood Production. 2019. Published online at OurWorldInData.org. Fish and seafood consumption per capita.

