# Supplementary material for: Haff Disease in Salvador, Brazil, 2016-2021: Attack rate and detection of toxin in fish samples collected during outbreaks and disease surveillance
Source: Lancet Reg Health Am. 2021 Nov 1;5:100092. doi: 10.1016/j.lana.2021.100092 (PMC9904022; doi:10.1016/j.lana.2021.100092)
Supplement: Supplementary file 1 [file mmc1.docx]

**Supplementary material.** Additional details on the analyses carried out in the fish samples.

**Arsenic**

*Method:*

Inductively coupled plasma mass spectrometry (ICP-MS) for quantification of total arsenic.

High performance liquid chromatography (HPLC) coupled to ICP-MS for arsenic speciation.

*Equipment:*

HPLC Infinity 1290 Agilent e ICP-MS 7900 Agilent

*Reference material used:*

Fish muscle ERM BB422

*Reference value:*

1.00 mg/kg by the ANVISA

Not determined by the FAO for fish samples.

*References:*

Batista BL, Nacano LR, De Souza SS, Barbosa F Jr. Rapid sample preparation procedure for As speciation in food samples by LC-ICP-MS. Food Addit Contam Part A Chem Anal Control Expo Risk Assess. 2012;29(5):780-8. doi: 10.1080/19440049.2011.645218. Epub 2012 Jan 18. PMID: 22257141.

Batista BL, Souza JM, De Souza SS, Barbosa F Jr. Speciation of arsenic in rice and estimation of daily intake of different arsenic species by Brazilians through rice consumption. J Hazard Mater. 2011 Jul 15;191(1-3):342-8. doi: 10.1016/j.jhazmat.2011.04.087. Epub 2011 Apr 27. PMID: 21601359.

Food and Agriculture Organization of the United Nations and World Health Organization. Codex Alimentarius – International Food Standards. General standard for contaminants and toxins in food and feed – CXS 193-1995. Adopted in 1995. Last revision in 2019. http://www.fao.org/fao-who-codexalimentarius/sh-proxy/en/?lnk=1&url=https%253A%252F%252Fworkspace.fao.org%252Fsites%252Fcodex%252FStandards%252FCXS%2B193-1995%252FCXS_193e.pdf

Resolução ANVISA RDC Nº 42 DE 29/08/2013. https://bvsms.saude.gov.br/bvs/saudelegis/anvisa/2013/rdc0042_29_08_2013.html

**Lead**

*Method:*

Inductively coupled plasma mass spectrometry (ICP-MS) for quantification of total arsenic.

*Reference value:*

0.30 mg/kg

*References:*

Resolução ANVISA RDC Nº 42 DE 29/08/2013. https://bvsms.saude.gov.br/bvs/saudelegis/anvisa/2013/rdc0042_29_08_2013.html

**Cadmium**

*Method:*

Inductively coupled plasma mass spectrometry (ICP-MS) for quantification of total arsenic.

*Reference value:*

0.05 mg/kg

*References:*

Resolução ANVISA RDC Nº 42 DE 29/08/2013. https://bvsms.saude.gov.br/bvs/saudelegis/anvisa/2013/rdc0042_29_08_2013.html

**Ciguatoxin**

*Method:*

Ciguatoxin analysis by LC-MS/MS and in vitro neuroblastoma cytotoxicity assay (N2a assay).

1. *Sample Preparation and Cytotoxicity Assay:*

Each sample was homogenized, and two 10 g sub-samples were taken from each sample for chemical extraction. Each sub-sample was extracted with acetone, and the resulting extract subjected to solvent partitioning and solid-phase extraction clean-up. A portion of each extract was examined for CFP-related toxins, and composite toxicity was assessed using an in vitro sodium channel-specific mouse neuroblastoma (N2a) cytotoxicity assay. Caribbean ciguatoxin-1 (C-CTX-1) was used as the standard.

1. *Liquid Chromatography-Mass Spectrometry:*

Sample extracts were analyzed for ciguatoxins using liquid chromatography-tandem mass spectrometry (LCMS/MS). The LC-MS/MS system consisted of an Agilent 1260 LC and Applied Biosystems MDS Sciex 4000 QTRAP mass spectrometer. LC-MS/MS analysis was performed in positive ion mode using multiple reaction monitoring (MRM) specific for ciguatoxins.

**Analysis for identification of fish species**

*Method:*

Species identification analysis was performed according to the FDA's Center for Food Safety and Applied Nutrition standard operating procedure: DNA Based Fish Identification (Barcoding) Method: Version 2: November 2011.

<https://www.fda.gov/Food/FoodScienceResearch/DNASeafoodIdentification/ucm237391.htm>

*References:*

Lee A. Weigt, Carole C. Baldwin, Amy Driskell, David G. Smith, Andrea Ormos, and Eric A. Reyier. Using DNA Barcoding to Assess Caribbean Reef Fish Biodiversity: Expanding Taxonomic and Geographic Coverage.

PLOS ONE 7 (7):e41059, 2012.

**Screening for palytoxin-like compounds**

*Method:*

LC-MS/MS

*Reference:*

Brissard C, Hervé F, Sibat M, Séchet V, Hess P, Amzil Z, et al. Characterization of ovatoxin-h, a new ovatoxin analog, and evaluation of chromatographic columns for ovatoxin analysis and purification. J. Chromatogr. A. 2015;1388, p. 87-101. https://doi.org/10.1016/j.chroma.2015.02.015.

**Sample toxicity**

*Method:*

Mouse bioassay for lipophilic biotoxins

*Reference:*

Riobó P, Paz B, Franco JM, Vázquez JA, Murado MA, Cacho E. Mouse bioassay for palytoxin. Specific symptoms and dose-response against dose-death time relationships. Food Chem. Toxicol. 2008;46, 2639–2647. https://doi.org/10.1016/j.fct.2008.04.020
